# Usability, acceptability, and cost of the SD BIOLINE Ov16 rapid diagnostic test for onchocerciasis surveillance in endemic communities in the middle belt of Ghana

**DOI:** 10.1371/journal.pntd.0012191

**Published:** 2025-08-29

**Authors:** Kenneth Bentum Otabil, María-Gloria Basáñez, Elizabeth Ameyaa, Michael Oppong, Prince Mensah, Richmond Gyasi-Ampofo, Emmanuel John Bart-Plange, Theophilus Nti Babae, Lydia Datsa, Andrews Agyapong Boakye, Michael Tawiah Yeboah, Prince Nyarko, Prince Charles Kudzordzi, Anabel Acheampong, Edwina Twum Blay, Henk DFH Schallig, Robert Colebunders

**Affiliations:** 1 Department of Biological Science, Consortium for Neglected Tropical Diseases and One Health, School of Sciences, University of Energy and Natural Resources, Sunyani, Bono Region, Ghana; 2 Faculty of Medicine and Health Sciences, Global Health Institute, University of Antwerp, Belgium; 3 Department of Infectious Disease Epidemiology, MRC Centre for Global Infectious Disease Analysis (MRC GIDA), and London Centre for Neglected Tropical Disease Research, School of Public Health, Imperial College London, London, United Kingdom; 4 Department of Medical Laboratory Science, School of Sciences, University of Energy and Natural Resources, Sunyani, Bono Region, Ghana; 5 Administration, Deo Gratias Medical Laboratories, Sunyani, Bono Region, Ghana; 6 Administration, Kintampo Health Research Centre, Kintampo, Bono East Region, Ghana; 7 Ghana Health Service, Regional Neglected Tropical Diseases (RNTD) Office, Regional Health Directorate, Sunyani, Bono Region, Ghana; 8 Department of Medical Microbiology, Academic Medical Centre, Experimental Parasitology Unit, Amsterdam University Medical Centres Amsterdam, Amsterdam, The Netherlands; 9 Amsterdam Institute for Global Health and Development, Amsterdam, The Netherlands; 10 Department of Tropical Disease Biology, Liverpool School of Tropical Medicine, Liverpool, United Kingdom; Cyprus International University: Uluslararasi Kibris Universitesi, CYPRUS

## Abstract

**Background:**

Previous studies in the Bono Region (middle belt) of Ghana have reported persistent *Onchocerca volvulus* infection and associated morbidities after nearly three decades of ivermectin treatment. This study aimed to assess the usability, acceptability, and cost of the Ov16 SD BIOLINE rapid diagnostic test (Ov16 RDT) in onchocerciasis surveillance activities in the middle belt of Ghana.

**Methodology:**

A cross-sectional study was conducted in 6 endemic communities in the Tain District and Wenchi Municipality. A total of 254 individuals (54% females; median age (range)=31 (5–83) years), agreed to participate in Ov16 RDT (100%), skin-snip microscopy (37%) and nodule palpation (100%). A total of 94 individuals participated in all three diagnostic tests, and post-test interviews were conducted with them, as well as with nine technicians. A cost analysis was also performed based on testing a hypothetical cohort of 400 individuals.

**Principal findings:**

Seropositivity of IgG4 antibodies against the Ov16 *O. volvulus* antigen was 23.6% (60/254, 95%CI = 18.8%–29.2%); microfilarial positivity 11.7% (11/94, 95%CI = 6.7%–19.8%) and nodule positivity 5.5% (14/254, 95%CI = 3.3%–9.0%). Female sex, age over 30 years, and farming occupation were all associated with higher odds of anti-Ov16 seropositivity. Among 5–9-year-olds, anti-Ov16 seropositivity was 11.1% (3/27), microfilarial positivity 23.1% (3/13) and nodule positivity 3.7% (1/27). Most participants and technicians preferred Ov16 RDT because of being less painful and invasive, easier to use and faster. Had 400 participants been tested, the total cost per individual would be US$24 (Ov16 RDT) and US$74 (skin-snip microscopy).

**Conclusions:**

Ov16 RDT is more acceptable and affordable (a third of the cost) compared to skin-snipping for surveillance activities in transmission hotspots in Ghana.

## Author summary

Onchocerciasis (River blindness) is a neglected tropical disease targeted by the World Health Organization for elimination of transmission in 12 endemic countries by 2030. There is a need for field-friendly, acceptable and affordable tools to monitor progress towards elimination. In Ghana, the SD BIOLINE Ov16 rapid diagnostic test (Ov16 RDT) has been used in several epidemiological surveys, but its usability, acceptability, and cost have not been assessed. We studied 6 endemic communities with persistent infection after nearly three decades of ivermectin treatment. The proportion of seropositivity by Ov16 RDT was twice the positivity of skin-snip microscopy positivity and four times the positivity of nodule-palpation positivity. The Ov 16 RDT was acceptable to both study participants and technicians because it was less painful and invasive, and yielded results more quickly. The cost of skin-snip microscopy would be thrice that of Ov16 RDT when testing 400 individuals. Ov16 RDT is more acceptable and less costly than skin-snipping for surveillance activities in transmission hotspots in Ghana.

## Introduction

Onchocerciasis is a severely debilitating, vector-borne neglected tropical disease (NTD) caused by infection with the filarial nematode *Onchocerca volvulus* and transmitted among humans by the bites of *Simulium* blackflies. Although the great majority (99%) of the cases are found in sub-Saharan Africa (SSA), the infection also occurs in Yemen and in two (Venezuela and Brazil) out of the six original endemic countries in Latin America [1]. In 2017, it was estimated that at least 220 million people required preventive chemotherapy against onchocerciasis, and according to the Global Burden of Disease (GBD) Study for that year, 14.6 million of those infected had skin disease and 1.15 million had vision loss [2]. In 2021, the GBD Study estimated that 19.6 (95% Uncertainty Interval, UI = 17.8–21.7) million people were infected, and that the disease was responsible for 1.26 (95%UI = 0.753–1.90) million disability-adjusted life-years (DALYs) [3].

Preventive chemotherapy relies on mass drug administration (MDA) delivered, in Africa, as community-directed treatment with ivermectin (CDTI), predominantly annually, but in several foci of some countries (such as Ethiopia, Ghana, Nigeria, Togo and Uganda), also 6-monthly (biannually). Ivermectin effectively kills microfilariae of *O. volvulus* and the rapid reduction in microfilarial load in the skin and eyes alleviates disease symptoms like skin lesions, itching, and visual impairment [4]. Ivermectin temporarily inhibits the release of new microfilariae by adult female worms (macrofilariae) leading to the transient decline in microfilarial density, however microfilaria production eventually resumes [5]. Additionally, repetitive doses of ivermectin have been known to exert a partial macrofilaricidal effect on the parasite, culminatively reducing the lifespan and fertility of adult female worm. The cumulative microfilaricidal and embryostatic effects of ivermectin on *O. volvulus* parasites are critical for interrupting the parasite’s life cycle and achieving onchocerciasis elimination [5]. Therefore, community-directed treatment with ivermectin (CDTI) must be sustained over many years to ensure the cumulative effects of the drug are sufficient to achieve elimination of *O. volvulus* transmission (EOT) [6]. With ivermectin MDA, EOT has been reported for some foci of Mali, Senegal, Nigeria and Sudan, without vector control [7]. In consequence, the World Health Organization (WHO) 2021–2030 NTD roadmap has proposed that 12 endemic countries be verified for EOT of onchocerciasis by 2030 [8].

The WHO has delineated three distinct phases in onchocerciasis elimination programmes: I) the treatment phase with CDTI, during which monitoring and evaluation are conducted periodically to ascertain progress towards transmission suppression and ultimately interruption; II) the post-treatment surveillance (PTS) phase, during which CDTI is no longer delivered but indicators of exposure and transmission are evaluated to confirm that EOT has been reached, and III) the post-elimination surveillance (PES) phase, to alert of potential resurgence of transmission or re-introduction of infection [9]. These distinct phases require different diagnostic tools that allow transitioning from one to the next. During phase I, and until Stop-MDA surveys are conducted, the gold standard for parasitological diagnosis and calculation of epidemiological indicators (microfilarial prevalence and load), has been the detection and enumeration of *O. volvulus* microfilariae by the so-called skin-snip microscopy method [10]. Skin-snip microscopy is highly specific provided that microfilarial morphometric characteristics are assessed, and able to detect active infection (as microfilariae are the embryonic progeny of reproductively active adult worms), but its sensitivity (based on two snips) decreases as microfilarial prevalence and load decline due to CDTI [11]. The procedure is also mildly invasive and resource-intensive, and its acceptability diminishes in communities where decades of skin-snipping have occurred [12].

Skin-snip Polymerase Chain Reaction (PCR) has been suggested to circumvent the problem of low sensitivity posed by dwindling skin microfilarial loads, to confirm the infection status of children seropositive for IgG4 antibodies against the Ov16 *O. volvulus* antigen, or when species-specific identification of microfilariae may be necessary [13]. However, it is not practical for large-scale community surveillance in SSA because it is expensive to purchase and operate and requires highly-skilled personnel. Serological, enzyme-linked immunosorbent assay (ELISA) tests based on the detection and quantification of IgG antibodies to the *O. volvulus* Ov16 recombinant antigen have shown, in longitudinal human and chimpanzee studies, that anti-Ov16 seropositivity may precede skin-snip positivity, providing a marker of exposure to early infection stages [14]. Other studies, also in chimpanzees, have indicated that IgG4 seroconversion against Ov16 coincides with the onset of patency (detectable skin microfilariae) [15], but as its duration may exceed that of patent infection, anti-Ov16 seropositivity may not yield a useful marker of active infection. Besides, ELISA-based serological tests also pose challenges including the need for laboratory analysis and the lack of a standardized, commercially-available Ov16 ELISA test, resulting in marked inter-laboratory variation [12]. A field-friendly, rapid diagnostic test (RDT) with the potential for integration into current onchocerciases surveillance activities in endemic countries, was developed and made commercially available (SD BIOLINE Onchocerciasis IgG4 rapid test) [16,17]. The performance of this Ov16 RDT has been evaluated in the field and current research priorities focus on operational and implementation research to demonstrate its usefulness, particularly in communities with low infection prevalence owing to many years of CDTI [18].

In Ghana, ivermectin MDA has been implemented for more than 30 years, having started in the late 1980s [19]. In 2009, a nationwide rapid epidemiological mapping of onchocerciasis (REMO) evaluation was conducted to provide updated information on the distribution of the disease. Areas with an infection (palpable nodule) prevalence in adult male samples above 20%, were allocated to a biannual treatment frequency, also considering a buffer zone of 20 km around these CDTI priority areas [20]. Forty-four districts were classified as meso- and hyperendemic and have received biannual MDA since 2010. Forty-one districts, which were re-classified as of low endemicity and were already on MDA before the REMO exercise, continued to receive annual MDA. From 2015 to 2020, efforts were intensified to accelerate the shift from onchocerciasis control to EOT. An operational threshold of microfilarial prevalence <1% was adopted, the implementation unit changed from community to sub-district, and the geographical and (reported) therapeutic coverage increased substantially. In 2017, an impact assessment survey was carried out using Ov16 serology in children aged <10 years and skin-snip microscopy in adults aged ≥20 years [21]. Together with historical data collected by the Onchocerciasis Control Programme in West Africa (OCP), the impact assessment helped the national NTD Programme to formulate strategies to reach EOT. Delineation epidemiological surveys were conducted in 50 hypoendemic districts that had never received ivermectin (not having been previously prioritized for control but in need of re-assessment for elimination), and treatment strategies and management decisions were redefined [20]. Currently, 137 districts require or are under biannual CDTI [21].

Previous studies in the Bono region of Ghana, in which some communities had been under biannual CDTI since 2010, revealed that *O. volvulus* infection and associated cutaneous and ocular morbidities persisted despite 27 years of treatment [21,22], and that despite high levels of reported coverage [22], treatment adherence was relatively poor [23]. Although such communities may not be ready to move from the treatment phase to Stop-MDA surveys or may require alternative interventions to accelerate progress [24], it is important to understand the role of different diagnostics for surveillance activities.

As Ghana transits from the treatment phase to the next stages of the EOT pathway in some endemic areas, and to evaluate progress towards suppression and interruption of transmission, the suitability of current diagnostic tools to accompany such transition needs to be ascertained. Whilst studies from other countries have investigated the acceptability, usability, and cost of the Ov16 RDT [12], no such study has been conducted in Ghana. Therefore, this study aimed to assess the diagnostic attributes, technician and community acceptability, usability, and cost of the Ov16 RDT in onchocerciasis surveillance activities in Ghana.

## Materials and methods

### Ethical considerations

Ethical clearance for this study was obtained from the Committee for Human Research and Ethics of the University of Energy and Natural Resources in Sunyani, Ghana, West Africa (approval number: CHRE/AP/012/021). The study was explained to the parents/guardians and school-age children in the local dialect (*Twi*). Informed written consent for children less than 18 years of age was given by their parents/guardians. Children gave oral assent to confirm their willingness to participate in the study. Participants were informed that they had the option of withdrawing at any stage of the study, without having to give any reasons, and without any consequences following withdrawal.

### Definition of terms

The study focused on three concepts: usability, acceptability, and cost. Usability refers to the extent a diagnostic test (in this context the Ov16 RDT and skin snip microscopy) can be used effectively and efficiently to achieve its intended purpose. In the context of onchocerciasis surveillance, the primary purpose of the Ov16 RDT is to detect ongoing transmission by testing children aged 5–9 years as a proxy indicator [8]. Acceptability evaluates whether a diagnostic test meets the needs and expectations of the users or stakeholders. In this study, stakeholders included both community members in endemic communities, and technicians performing the test. Cost refers to the total expenditure associated with conducting a diagnoistic test, including both direct and indirect costs. This includes the cost of materials, equipment, labour, and other operational expenses.

### Training to ensure quality of diagnostic tests

To ensure the quality of the diagnostic testing, we organized a comprehensive training for all Ov16 RDT performing technicians. Technicians were trained on the proper use and storage of the Ov16 RDT, essential testing guidelines and precautions, the test procedure and accurate interpretation of results. Key training materials included the Bench Aid for the Onchocerciasis Ov16 Rapid Diagnostic Test [25] and the manufacturer’s product insert.

### Study area

**Fig 1:**
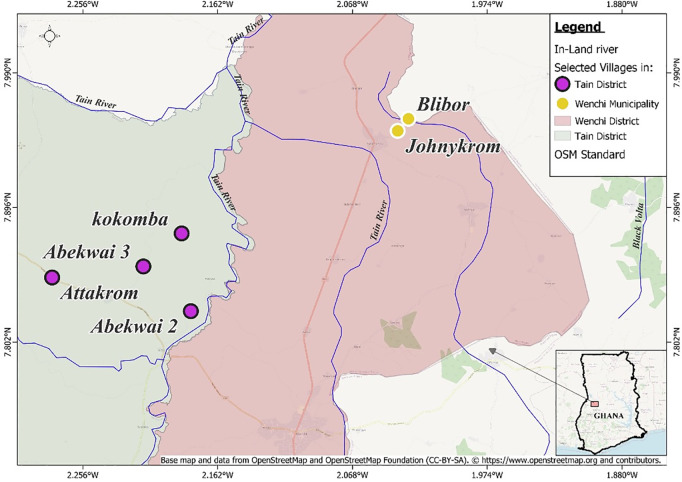
Map of study communities in the Tain District and Wenchi Municipality, Bono Region, Ghana. The light pink area to the right of the study area represents the Nkoranza North District. The study communities fall within the so-called transitional zone with mainly savannah type vegetation. The map data used in this document is attributable to OpenStreetMap contributors and is licensed under the Open Database License (ODbL). More information can be found at https://www.openstreetmap.org/copyright.

The study was performed in six communities, namely, four communities in the Tain District (Abekwai 2, Abekwai 3, Attakrom and Kokomba) and two communities in the Wenchi Municipality (Blibor and Jonnykrom/Adamakrom), in the Bono Region of Ghana. Fig 1 shows the locations of the study villages. The communities were selected based on the following criteria informed by previous studies [21,22]: i) they had persistent endemicity for *O. volvulus* infection, albeit with relatively low prevalence; ii) they had received ivermectin MDA for at least 20 years and biannual CDTI for about 10, and iii) they were situated close to fast-flowing rivers (the Subin, the Tain, or their tributaries) where the blackfly vectors breed. These criteria make the study communities representative of other onchocerciasis hotspots, which continue to exhibit persistent microfilarial infections and active blackfly breeding despite decades of CDTI and vector control interventions [26].

### Study design and recruitment

The study was conducted between June and August 2021. This was a cross-sectional study, using both quantitative and qualitative approaches. The survey assessed the onchocerciasis endemicity using Ov16 testing and skin-snip microscopy, and evaluated the usability, acceptability and cost of those two methods. The assessment was conducted through the following steps:

- Community mobilization and sensitization
- Sociodemographic Questionnaire administration
- Nodule palpation
- Ov16 RDT testing
- Skin-snip microscopy
- Qualitative assessment of participants and technicians through in-depth interviews

Details of these steps are provided below.

### Community mobilization and sensitization

One or two weeks before the surveys were conducted, announcements were made in the study communities through the community information centres or with the help of the gong-gong (local means of informing the community members by moving the gong-gong and periodically sounding it). Residents were informed to gather at selected community centres. After the meeting, the study was explained in English and then in the local Twi language in front of the village elders and community members at a designated meeting point. All volunteers aged ≥5years were invited to participate in the study and were recruited once they agreed and consented to it as described above

### Sociodemographic questionnaire administration

Participants were asked to state their sex and age, and respond to questions regarding whether they had been born in the village (yes or no); their duration of residence in the community (<1 year or ≥1 year); whether they lived close to a stream (yes or no), visited a stream (yes or no) and how often (every day or less than everyday), and their occupation (farming or non-farming), with the aim to obtain information indicative of exposure to explore its association with anti-Ov16 status (positive or negative).

### Nodule palpation

All participants were examined for palpable nodules by trained members of the medical team (including a doctor, nurse, and laboratory technicians) with experience in detecting onchocercal nodules (onchocercomas). Palpations were done on bony protuberances of the ribs, iliac crests, sacrum, and upper leg, but not at locations (neck, axillae, inguinal) typical for lymph nodes. Nodules were defined as ‘suspected’ onchocercomas (mass) (i.e., not confirmed by dissection or biopsy) which were firm, often flattened or bean-shaped, usually movable, non-tender and up to several centimetres in diameter.

### Ov16 RDT testing

The deployment of the IgG4-based SD BIOLINE rapid diagnostic test (Ov16 RDT) followed the manufacturer’s instructions (Standard Diagnostics, Yongin-si, Gyeonggi-do, South Korea) and quality-assurance procedures. Briefly, the test cassette was labelled with the participant’s unique identification number. A finger (the middle or ring finger on the left hand) of the participant was selected, cleaned with an alcohol swab, and pricked with the lancet provided with the kit. The blood was then collected using a capillary tube and the blood was placed in the specimen well of the cassette. Four drops of assay diluent were also placed in the assay diluent well. RDT results were read after 30 minutes [12].

### Skin-snip microscopy

Skin snipping was performed as described in our previous studies [21,22]. Briefly, the left and right iliac crests of each participant were disinfected using alcohol swabs. Holth corneoscleral punches were used to take about 2mg of bloodless skin snips each from the left and right iliac crests. The snips were placed separately in microtitre wells containing 100 µl normal saline (NaCl 0.85%) solution and incubated at room temperature for 24 hours. After this period, the incubation medium was pipetted onto a microscope slide and examined using, firstly, a x10 and subsequently a x40 objective lens. If microfilariae (mf) had emerged, the participant was recorded as skin-snip positive. However, we did not confirm the identification of *O. volvulus* microfilariae using morphological methods (e.g., fixation and staining for microscopic examination) or molecular techniques (e.g., PCR analysis).

### Qualitative assessment of participants and technicians through in-depth interviews

To evaluate the usability and acceptability of the RDT, all nine RDT technicians who also had experience in skin-snip microscopy, and 94 community residents who participated in all three tests (RDT, skin-snip microscopy and nodule palpation) were interviewed. The interview guide (S1 Text) was developed along the themes of preference and reasons for preference by participants and technicians regarding the use of Ov16 RDT for surveillance following [12]. The interviews were recorded using an audio recorder on Android phones and transcribed *verbatim*. Interview data were coded using content analysis based on key themes from the semi-structured interviews as done in [12].

### Economic costs

To evaluate the cost associated with the implementation of onchocerciasis surveillance programmes in Ghana using Ov16 RDT and skin-snip microscopy, a costing analysis was done based on 400 participants as the basis for our calculations to enable comparison of costs for each test without the effect of the numbers actually tested in any given survey (see cost breakdown in S2 Text). Briefly, the value of volunteers’ time as well as the surveillance costs were computed using the ‘ingredient’ approach, multiplying the input prices by the number of inputs used [27]. The major input prices for this analysis were the cost of each: a) Ov16 RDT (US$2.82 per cassette); b) Holth corneoscleral punch (US$423.5 per punch), and c) optical microscope (US$1,694). In addition, costs were captured for all activities conducted during the surveillance activity, including training, fieldwork, and data reporting. Fieldwork cost categories were further split into transport and lodging, supplies, devices and instruments, and labour. An exchange rate of GHS7.08 (cedis) per US$ was used in agreement with the rate for 2022 [28].

### Sample size and statistical analysis

A formal sample size was not calculated a priori for the study sites; instead a convenience sampling approach was used, yielding an average sample size equivalent to 12% (range = 5%–33%) of the respective community populations (Table 1). Nonetheless, we aimed to achieve at least 30 participants per community in line with typical thresholds reported in onchocerciasis epidemiological studies [29].

**Table 1:** Number of participating communities, their sex composition, median age, and numbers examined for each onchocerciasis diagnostic.

Community	Recruited/ Population (%)	Sex^†^		Median age^ǂ^ (range) (Years)	No. Tested		
		Females (%)	Males (%)		Ov16 RDT (%)	Skin snip (%)	Nodules (%)
**Tain District**							
Abekwai 2	30/150 (20.0%)	17 (56.7%)	13 (43.3%)	22 (5–61)	30 (100%)	11 (36.7%)	30 (100%)
Abekwai 3	60/700 (8.6%)	29 (48.3%)	31 (51.7%)	30 (5–83)	60 (100%)	23 (38.3%)	60 (100%)
Attakrom	41/800 (5.1%)	21 (52.5%)	19^†^ (47.5%)	47 (9–80)	41 (100%)	15 (36.6%)	41 (100%)
Kokomba	40/120 (33.3%)	24 (60.0%)	16 (40.0%)	25 (7–60)	40 (100%)	16 (40.0%)	40 (100%)
**Wenchi Municipality**							
Blibor	47/240 (19.6%)	18 (38.3%)	29 (61.7%)	27 (7–80)	47 (100%)	12 (25.5%)	47 (100%)
Johnykrom/ Adamakrom	36/170 (21.2%)	27 (75.0%)	9 (25.0%)	41 (6–72)	36 (100%)	17 (47.2%)	36 (100%)
**Total**							
	254/2,180 (11.7%)	136 (53.8%)	117 (46.3%)	31 (5–83)	254 (100%)	94 (37.0%)	254 (100%)

†Of the 254 participants, sex was recorded for 253 (sex missing for one participant in Attakrom). ^**ǂ**^Age was recorded for 242 participants (age missing for one participant in each of Abekwai 2 and Abekwai 3, four participants in Attakrom, three in Kokomba and three in Johnykrom).

Data were entered in purposely designed MS Excel spreadsheets (one for the epidemiology results and another for the cost menus). Analyses were conducted using Jamovi Desktop (version 2.3.19) and GraphPad Prism 8 for macOS (version 8.2.1). Descriptive statistical analysis was performed first to observe the characteristics of the variables. The main statistical analysis consisted of univariable and multivariable logistic regression with crude odds ratio (AOR) and adjusted odds ratio (AOR), respectively, to identify factors associated with Ov16 seropositivity in the study communities. When reporting proportions, 95%CIs were calculated using the Wilson score method [30]. Proportions were compared by assessing their 95%CIs or by using the z-statistic where appropriate (non-paired data), in which case asymptotic 95%CIs are reported [31]. The significance level for all analyses was set at 0.05.

## Results

Of the total of 254 individuals, there were 253 with recorded sex, consisting of 136 (53.8%) females and 117 (46.3%) males. Age was recorded for 242 individuals, with a median age of 31 (range = 5–83) years (Table 1).

A total of 254 individuals (100%) consented to the Ov16 RDT, 94 (37%) to skin-snip microscopy, and 254 (100%) to nodule palpation. Consequently, 94 participants underwent all three tests. Among this subset, the median age was 31.0 years (range = 5–83), 53.8% were female, 70.5% were farmers and 52.9% had lived in the community for more than one year.

The proportion of the population for whom microfilaridermia was assessed ranged from 26% to 47%.

Of the 254 individuals tested for Ov16 RDT, 60 (23.6%, 95%CI = 18.8%–29.2%) were seropositive, compared to 11 (11.7%, 95%CI = 6.7%–19.8%) microfilaria-positive individuals out of the 94 with skin-snip results (z-value = 2.5; P-value = 0.0143). For palpable nodules, 14 out of 254 individuals examined were positive (5.5%, 95%CI = 3.3%–9.0%). The positivity of palpable nodules was only marginally significantly different from that of microfilaridermia (z-value = 2, P-value = 0.0471), but highly significantly different from that of anti-Ov16 seroprevalence (z-value = 5.8, P-value <0.0001).

The distribution of Ov16 seropositivity, skin snip positivity and nodule palpation result based by dichotomised sociodemographic variables is presented in Table 2. Being female, over 30 years of age, being a farmer, living in proximity to streams and frequently visiting streams were all associated with higher odds of being anti-Ov16 seropositive.

**Table 2:** Positivity rates of the three onchocerciasis diagnostic tests and multivariable analysis of associated sociodemographic variables.

Categories	Anti-Ov16 positive, n (%) [95% CI]	Skin snip positive, n (%, 95% CI)	Nodules n, (%) [95% CI]	COR [95% CI]	AOR [95% CI]
**Overall**	60/254 (23.6%) [18.8–29.2]	11/94 (11.7%) [6.7–19.8]	14/254 (5.5%) [3.3–9.0]	–	–
**Community**					
Abekwae 2	10/30 (33.3%) [19.2–51.2]	1/11 (9.1%) [1.6–37.7]	3/30 (10.0%) [3.5–25.6]	Reference	Reference
Abekwae 3	15/60 (25.0%) [15.8–37.2]	3/23 (13.1%) [4.5–32.1]	7/60 (11.7%) [5.8–22.2]	0.667 [0.256–1.738]	0.671[0.209–2.157]
Attakrom	12/41 (29.3%) [17.6–44.5]	1/15 (6.7%) [1.2–29.8]	0/41 (0.0%) [0.0–8.6]	0.828 [0.300–2.282]	1.846 [0.068–50.000]
Kokomba	4/40 (10.0%) [4.0–23.1]	4/16 (25.0%) [10.2–49.5]	1/40 (2.5%) [0.4–12.9]	0.222 [0.061–0.801]	0.143 [0.030–0.673]
Blibor	5/47 (10.6%) [4.6–22.6]	1/12 (8.3%) [1.5–35.4]	1/47 (2.1%) [0.3–11.1]	0.238 [0.072–0.789]	0.197 [0.051–0.764]
Johnnykrom/ Adamakrom	14/36 (38.9%) [24.8–55.1]	1/17 (5.9%) [1.1–27.0]	2/36 (5.6%) [1.1–27.0]	1.273 [0.462–3.503]	0.508 [0.141–1.822]
**Sex category**					
Male	17/117 (14.5%) [9.3–22.0]	5/41 (12.2%) [5.3–25.5]	8/117 (6.8%) [3.5–12.9]	Reference	Reference
Female	43/136 (31.6%) [24.4–39.9]	6/53 (11.3%) [5.3–22.6]	6/136 (4.4%) [2.0–9.3]	2.720 [1.451–5.100]	1.777 [0.826–3.778]
**Age**					
<30 years	13/114 (11.4%) [6.8–18.5]	7/44 (15.9%) [7.9–29.4]	4/114 (3.5%) [1.4–8.7]	Reference	Reference
≥30 years	42/128 (32.8%) [25.3–41.3]	4/47 (8.5%) [3.4–19.9]	10/128 (7.8%) [4.3–13.8]	3.794 [1.912–7.529]	3.610 [1.205–10.813]
**Occupation**					
Non-Farming	9/74 (12.2%) [6.5–21.5]	5/28 (17.9%) [7.9–35.6]	4/74 (5.4%) [2.1–13.1]	Reference	Reference
Farming	50/177 (28.2%) [22.1–35.3]	6/65 (9.2%) [4.3–18.7]	10/177 (5.6%) [3.1–10.1]	2.843 [1.316–6.141]	1.396 [0.376–5.180]
**Duration of stay in the village**					
Born in village and <1 year	23/115 (20.0%) [13.7–28.2]	6/45 (13.3%) [6.3–26.2]	4/115 (3.5%) [1.4–8.6]	Reference	Reference
≥1 year	31/129 (24.0%) [17.5–32.1]	5/46 (10.9%) [4.7–23.0]	10/129 (7.8%) [4.3–13.7]	1.265 [0.688–2.328]	0.815 [0.386–1.721]
**Proximity to stream**					
No	11/39 (28.2%) [16.5–43.8]	1/13 (7.7%) [1.4–33.3]	0/39 (0.0%) [0.0–9.0]	Reference	Reference
Yes	49/215 (22.8%) [17.7–28.9]	10/81 (12.3%) [6.9–21.3]	14/215 (6.5%) [3.9–10.6]	0.751 [0.349–1.618]	3.254 [0.136–78.123]
**Visit stream**					
**No**	16/63 (25.4%) [16.3–37.3]	2/23 (8.7%) [2.4–26.8]	0/63 (0.0%) [0.0–5.8]	Reference	Reference
**Yes**	40/173 (23.1%) [17.5–30.0]	8/63 (12.7%) [6.6–23.1]	13/173 (7.5%) [4.4–12.4]	0.883 [0.453–1.724]	1.305 [0.335–5.086]

COR: Crude Odds ratio, AOR: Adjusted Odds Ratio.

Among individuals who tested anti-Ov16 seropositive, 10.5% (95%CI = 2.9%–31.4%) also had a positive skin snip, and 11.7% (95%CI = 5.8%–22.2%) presented with palpable onchocercomas.

Of the 17 participants who were anti-Ov16 positive but skin-snip negative, 13 (76.5%) were female and all 17 had taken ivermectin in the previous year. No participant tested positive by all three tests.

The results of the age and sex distribution of anti-Ov16 RDT, skin-snip and palpable nodule positivity is provided in S1 Table.

In the 5–9 age group, 3/27 children (11.1%, 95%CI = 3.9%–28.1%) were anti-Ov16 seropositive, 3/13 children (23.1%, 95%CI = 8.2%–50.3%) skin-snip positive, and 1/27 (3.7%, 95%CI = 0.7%–18.3%) nodule positive.

### Acceptability of Ov16 RDT, skin-snip microscopy and nodule palpation

The 94 individuals who participated in all three tests were interviewed after the diagnostics were completed (exit questionnaire, S1 Text). Most of the participants (71/94 = 75.5%) preferred the Ov16 RDT to skin-snip microscopy citing reasons such as the Ov16 RDT being less painful, less invasive and providing results within 30–40 minutes, rather than waiting until the next field visit to learn the result of the skin snip testing.

*“This test (Ov16 RDT) is better! It is less painful than the one where you (health personnel) ‘cut the buttocks’ (skin snip)” (Female participant, Abekwai 2).*

*“I don’t normally see the need to participate in the test where you take some skin from my buttocks. A lot of times, you (health personnel) just cut the skin and we don’t hear anything about our results. I like this new one (Ov16 RDT) as I got to know my results immediately” (Male participant, Abekwai 3).*

Despite the majority’s preference for Ov16 RDTs, 34% (32/94) remarked that they were ‘okay’ with skin-snip microscopy and were willing to ‘endure’ the pain, as long as they were convinced that undergoing the test would improve their health and wellbeing. This proportion of respondents was in agreement with the 37% of the recruited individuals who agreed to be skin-snipped (Table 1).

*“For me, I am okay if you (technician) cut skin on my buttocks (skin snip), as long as it will help towards treating me early. The pain is only temporary, good health is what matters”* (Adult male, Attakrom).

However, 20% (19/94) of participants detested the skin-snip method and stated that they would not engage in any future surveys involving this method. The major reasons for this included the painful nature of skin-snipping and its invasiveness. Some participants also argued that they experienced itching, developed a rash, or suffered from other allergic reactions after they had been skin-snipped:

*“For me, I will run away if you (technician) come here again with that your scissors, it is too painful” (Female participant, Johnykrom).*

Regarding the use of nodule palpation, 98% (92/94) of the study participants were ‘okay’ with it, with 18% (17/94) preferring to be examined for nodules by a person of the same sex, and 2% (2/94) not being at all comfortable with being palpated for nodules due to religious reasons:

“*As for me, I don’t like it when the female doctor is touching parts of my body as she will see my nakedness” (Adult male, Blibor).*

### Usability and acceptability of the Ov16 RDT among technicians in the study

The study interviewed all nine technicians who performed the Ov16 RDTs and who were also experienced in skin-snip microscopy, with 89% (8/9) preferring the former test over the latter. The reasons for this included that it required less effort and training and met with less resistance from the survey participants on the account of being less painful and less invasive. The rapid turnaround time of the Ov16 RDT results (within 30–40 minutes from sampling to results) as opposed to more than 24 hours for skin-snip microscopy further increased the willingness of the community to take part in surveillance activities. The technicians also liked the fact that children were able to participate in the serological survey. Most of the technicians also indicated that they trusted the results from the Ov16 RDT as it tends to lend itself to fewer human errors. When prompted on the challenges with the use of the Ov16 RDT, most technicians referred to the fact that being antibody-based, the test could not differentiate between past and current infections. They therefore advocated for both tests; first Ov16 RDT for indication to exposure, followed by confirmation of active infection with skin-snip microscopy when necessary. Nevertheless, all technicians remarked that they would prefer to use Ov16 RDTs in future surveillance activities.

### Cost analysis for Ov16 RDT and skin-snip microscopy

*Time considerations*. The training of nine technicians for the Ov16 RDT took a maximum of two hours (120 minutes) in contrast to 14 days (6,720 minutes for an 8-hour daily schedule) for the training of five technicians on skin-snip microscopy. The Ov16 RDT took approximately 10 minutes to take a sample from each participant (labelling the cassette, selecting, and cleaning a finger, pricking the finger and placing the blood and the assay diluent in the cassette) and 30–40 minutes to get and communicate the results (30 minutes to read the results from the cassette and 10–20 minutes to record such results and convey them to participants). By contrast, skin-snip microscopy took about 30 minutes for sampling from each participant (cleaning left and right iliac crests with alcohol swabs, taking the skin snips with disinfected punches, placing the snips in labelled microtitre plate wells containing incubation medium, recording participant details), 24 hours for the incubation of the snips and about 20 minutes to pipette and observe the incubation medium under a microscope under two magnifications, for a total of 24 hours and 50 minutes (1,490 minutes) to obtain the results. A common concern was that the participants of skin-snip microscopy typically never get to know their results or at best get to know them only during the next field visit, which can take weeks or months. These have cost implications for both Ov16 RDT and skin-snip microscopy tests.

**Fig 2:**
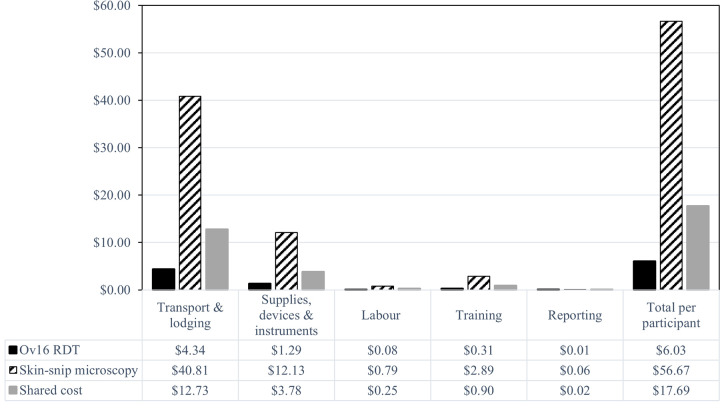
Cost analysis per Ov16 RDT and skin-snip microscopy for 400 individuals tested for each diagnostic.

*Cost considerations*. The total cost for the activities targeting 400 participants was estimated at US$32,156. The cost was apportioned to Ov16 RDT, skin-snip microscopy and shared cost. The costs incurred for Ov16 RDT and skin-snip microscopy test and activities were US$2,412 (7.5%) and US$22,670 (70.5%), respectively. The shared cost incurred accounted for 22% of the total cost of the study (US$7,074). Within the total cost, 94.8% (US$30,484) corresponded to fieldwork cost, comprised of transport and lodging (72.0%), supplies, devices and instruments (21.4%), and labour cost (1.4%). Training and reporting costs accounted for 5.1% (US$1,640) and 0.1% (US$32) of the total cost, respectively. The total cost per participant of the Ov16 RDT was US$ 23.72 (US$6.03 for test-specific costs and US$17.69 for shared costs); the cost of skin-snip microscopy was US$74.36 (US$56.67 for test-specific costs and US$17.69 for shared costs). The cost per participant of deploying Ov16 RDT and skin-snip microscopy testing based on 400 participants and split by category is presented in Fig 2.

Proportionally reducing costs according to the numbers tested in our study (254 for Ov16 RDT and 94 for skin-snip microscopy) and adjusting for fixed inputs, the total cost of the activity was estimated at US$24,708 for the 6 communities surveyed, with a net cost per participant of US$27 for Ov16 RDT and US$207 for skin-snip microscopy, highlighting the impact of the volume effect [10].

## Discussion

This study aimed to determine the usability, acceptability, and cost of the Ov16 RDT for onchocerciasis surveillance in endemic communities in the middle belt of Ghana, particularly in the Tain District and Wenchi Municipality where it has been reported that onchocerciasis persists after 27 years of ivermectin MDA [21,22]. The positivity of anti-Ov16 IgG4 antibodies among study participants was 24%, the microfilarial positivity was 12% and the nodule prevalence was 6%. Female sex, age over 30 years, being a farmer, residence near streams and frequent stream exposure were all associated with higher odds of anti-Ov16 seropositivity (Table 2). Among the anti-Ov16 seropositives, there was a significantly higher proportion of females, those aged 30 years or older, living near streams, visiting such streams, and engaged in farming. However, the proportions of those presenting skin microfilariae or palpable nodules were significantly lower.

Since the study communities have received ivermectin MDA for nearly three decades, those aged ≥30 years at the time of our study would have been exposed as children to baseline levels of transmission. We have previously reported that the study area was initially mesoendemic, with a baseline microfilarial positivity of 41% to 48% [21]. This is broadly in agreement with the 33% seropositivity we found among those 30 years or older. Of the 126 females sampled, more than half (61%) were aged ≥30 years, whereas of the 116 males sampled, less than half (44%) belonged to this age group; nearly 100% of those 30 years of age or older were farmers (76/77 females and 50/51 males).

As the blackfly vectors breed in fast-flowing rivers and streams [1], it is perhaps not surprising that those living near or visiting such water bodies were more frequently represented among seropositive individuals, but contrary to expectation the proportion of those visiting streams daily was lower than that of those visiting less often. Farming can be another activity that increases exposure to blackfly bites, and historically, agricultural communities are known to have been highly endemic for onchocerciasis as farms are often situated near rivers that provide water for crops [32]. As blackflies are outdoor and diurnal biters, farmers working outdoors for several hours of the day may have an increased risk of exposure. In the Tombel Health District, Southwest region of Cameroon, and after 15 years of CDTI, Nyagang et al. [33] reported higher onchocerciasis prevalence in those aged more than 60 years, who were mostly farmers. The greatest prevalence of infection (2.1%) was among farmers [33]. A study in Southwest Ethiopia, also after 15 years of CDTI, reported that individuals aged ≥35 years had a microfilarial prevalence of 15.7% compared to 1.4% in those aged 15–24-years (no children under 15 were examined), and that approximately 60% of the participants were farmers, of whom 9% were microfilaria-positive [34]. The authors stated that adults were engaged in outdoor activities, increasing their exposure to blackfly bites [34].

Although anti-Ov16 IgG4 seroprevalence levels, particularly in the young, have been considered to indicate exposure to infection, knowledge of the parasite stage(s) that elicit IgG4 antibody seroconversion to the Ov16 antigen, as well as of the dynamics of seroconversion and potential seroreversion or antibody decay under transmission interruption remain poorly understood. A recent study by Cama et al. [35], estimated the antibody response half-life to be 3.3 (2.7–4.1) years, indicating that the majority of seropositive people will serorevert over time following interruption of transmission. Willen et al. [36,37] reported, using data from Ghana, on the development of an IgG-based immunoassay to quantify antibodies to *Simulium damnosum* sensu lato saliva that, if combined with assays to detect past or current exposure to *O. volvulus* could help to investigate exposure to blackfly bites and to the parasite.

**Fig 3:**
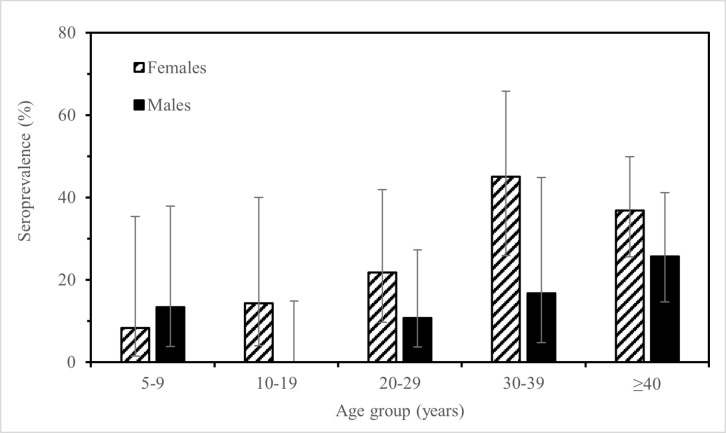
Anti-Ov16 seropositivity by age group and sex among 242 participants with recorded age and tested by Ov16 RDT. Hatched bars: Females, black bars: males, error bars: 95% confidence intervals.

In our study, anti-Ov16, skin-snip and nodule positivity were generally higher among those aged 30 years or older (except for microfilarial prevalence, which was lowest in those ≥40 years of age). Since only 94/254 (37%) of the participants agreed to be skin snipped, with complete data on age and sex for 91 of these, our results need to be interpreted with caution. Notwithstanding our small sample sizes, 11%, 23% and 4% of the 5–9-year olds examined were positive for IgG4 antibodies against Ov16, microfilaridermia and onchocercomas, respectively (S1 Table). As the nodule-positive child was aged 5 and the pre-patent period (from infection to detectable microfilaridermia) of *O. volvulus* has been estimated to range between 1 and 3 years [38], we can infer that this child became infected when he was 3–4 years of age, underscoring the occurrence of ongoing transmission in the study villages. Although anti-Ov16 seroprevalence by age was generally higher for females compared to males (except for the 5–9-year age group), sample sizes were small, leading to wide 95%CIs (Fig 3), precluding us from drawing firm conclusions about sex- and age-specific exposure patterns.

There were differences in positivity of anti-Ov16 and skin-snip positivity. This is likely explained by the fact that treatment leads to a reduction in skin microfilariae but antibodies to Ov16 may remain for much longer [12], with the levels of anti-Ov16 and skin-snip positivity becoming increasingly disassociated as treatment progresses. The finding of low anti-Ov16 seropositivity by RDT in skin-snip positive individuals is not totally unexpected, as it has been documented elsewhere [39]. Two factors may play a role; firstly, a proportion of infected individuals may not be able to mount an IgG4 antibody response to the Ov16 antigen (~20% has been reported in some studies, but between 5–10% in others [39–41]). Cross-reaction of Ov16 antibodies with other filarial worms is unlikely to influence the findings as only cross-reaction with *Mansonella ozzardi*, which is not present in Africa, has been reported [42]. Secondly, microfilariae of *Mansonella* spp. (rather than of *O. volvulus*) could potentially be present in some skin snips [43]. However, microfilariae of *M. perstans* are blood-dwelling and hence unlikely to have contaminated the bloodless skin snips we endeavoured to take [43]. Microfilariae of *M. streptocerca* are skin-dwelling, but the distribution of this parasite is confined to tropical rainforest areas [43], whereas our study site is mostly savannah (see Fig 1). Therefore, it is unlikely that the presence of *Mansonella* spp. microfilariae explains the discrepancy between skin-snip and anti-Ov16 positivity by RDT. Our results agree with reports of low Ov16 RDT sensitivity in areas with long-term treatment [39].

An interesting observation from our study was that the proportion of nodule positivity was about half that of skin snip positivity which was also about half the proportion of Ov16 positivity. This pattern warrants further investigation, as it may have practical implications for control programmes. If consistent it could enable the estimation of one diagnostic outcome (e.g., Ov16 seropositivity, skin snip positivity, or nodule prevalence) based on knowledge of another, thereby informing surveillance and decision-making in onchocerciasis-endemic areas.

We acknowledge that the WHO guidelines for stopping MDA and verifying elimination of transmission [29] do not advocate the use of anti-Ov16 serology to detect active infection or its use in all ages, but rather in those under 10 years of age using ELISA, with a seropositivity <0.1% at the upper 95% confidence upper bound in a sample of 2,000 children being taken as an indication of transmission interruption [29]. Modelling studies have investigated the applicability, to predict EOT outcomes, of this (and less stringent) threshold(s) and age group(s) under different assumptions of exposure and regulation of parasite abundance [44], and ongoing studies (some in Ghana) are being conducted to test such thresholds in the field.

We found that conducting Ov16 RDT testing in all ages provided useful information interpretable in the context of the duration of treatment in the area, as done by others in Mali and Tanzania [45,46]. Our findings also indicate that the study communities are not yet at the point of commencing Stop-MDA surveys despite nearly three decades of ivermectin MDA. We have documented the continued presence of *O. volvulus* infection and its associated clinical manifestations in these communities [21,22], and investigated factors influencing treatment adherence which may partly account for the persistence of transmission [23]. Entomological studies should also be undertaken in the study area to assess infection levels in simuliid population samples.

Studies done in other African settings with long-term CDTI (using the Ov16 RDT with whole blood) have reported, in the Ulanga district of Morogoro, Tanzania (an initially hyperendemic area), seroprevalence levels of 14% (27/191) in those aged 6–10 years and 33% (26/79) in those aged 11–12 after more than 20 years of CDTI [47]. In a formerly highly endemic area in the Mbam and Sanaga river valleys of Cameroon, Siewe Fodjo et al. [48] found an Ov16 seropositivity of 47% (68/145) in Bilomo (Centre Region) and 52% (13/25) in Kelleng (Littoral Region) among children aged 7–10 years after >13 years of CDTI. A factor likely involved in the persistence of onchocerciasis in this area is the very high (and perennial) biting rates of the blackfly vectors, as documented in other localities of the Mbam and Sanaga river systems, both historically and recently [49,50]. As our study area was originally mesoendemic, and annual biting rates in the region were of the order of 3,000–4,000 per person [21], it would be advisable to update entomological studies in the Tain District and Wenchi Municipality of Ghana to better understand the potential contribution of blackfly abundance, species composition and transmission intensity to persistent infection. A study in the Nkoranza North District (see Fig 1) documented highly seasonal transmission, with monthly biting rates of approximately 800 in the peak transmission season and 0–100 in other months of the year [51]. More recently, entomological studies have been conducted to better understand the role of *Simulium* vectors in sustaining onchocerciasis transmission close to our study areas [26]. Nditanchou and colleagues working in the Kwanware-Ottou transmission focus (within 5-10 km of our study communities) in Ghana found that the most productive blackfly breeding was within 5 km of Kwanware-Ottou. They also reported that blackfly daily biting rates were highest in Kwanware and Ottou, with 199 and 160 bites per day, respectively. Infection in blackflies was found only in Kwanware and Ottou, with infectivity rates of 5.9% (per 1000) and 6.7%, respectively [26]. These findings underscore the need for targeted vector control interventions in our study area and other transmission hotspots, using environmentally friendly approaches such as the “slash and clear” method [52].

In terms of diagnostic test acceptability, most participants preferred the Ov16 RDT to skin-snip microscopy and nodule palpation on account of its being less invasive, less painful, and more rapidly generating results that the participants could see *in situ*. These attributes of the RDT could be capitalised upon to increase the willingness of the population to participate in surveillance activities. Our findings are consistent with those of Dieye et al. [12] in south-eastern Senegal, who reported a greater willingness of participants to take part in onchocerciasis surveillance activities involving Ov16 RDT. Interestingly, the participation rates in Ov16 RDT (99.7%) and skin-snip microscopy (32.7%) in the Senegal study [12] are very similar to ours in Ghana (100% and 37%, respectively), suggesting that about a third of the population might be willing to participate in skin-snipping surveys. As endemic countries transit the path towards onchocerciasis EOT, it is increasingly important that the surveillance tools used in control and elimination programmes are both effective and acceptable. Residents of meso- and hyperendemic communities have typically had a long history of MDA treatment and are, therefore, increasingly apathetic towards control and surveillance activities, especially because the disease is no longer perceived as a major public health problem in some areas.

As mentioned, about a third of the individuals taking part in the ‘exit’ interviews remarked that although skin-snipping is painful, they would be willing to participate in future parasitological surveys using skin-snip microscopy. This was partly due to the research team members explaining to the participants the need for the test and the potential benefits to their health outcomes. These findings underscore the significant influence that surveillance staff can have on participation rates. They also highlight the importance of incorporating targeted education in NTD programmes to ensure that community members understand the rationale and value of the diagnostic tools used in surveillance, thereby fostering greater acceptance and participation.

The Ov16 RDT was also generally preferred among the technicians involved owing to its being relatively simple and easy to use, quick turnaround and potential for increasing community participation in future surveys, in agreement with the study in Senegal [12]. However, the technicians in our study were aware of the Ov16 RDT limitations concerning its inability to distinguish between past and present exposure/infection.

For a total of 400 individuals, the total cost per participant was estimated at US$24 for Ov16 RDT and US$74 for skin-snip microscopy, the former being approximately a third of the cost of the latter. The difference in cost between the two tests per individual (US$50) was mainly driven by differences in transport and lodging, supplies, devices and instruments, labour and training. Regarding training requirements, Ov16 RDT required a relatively short time (approximated 2 hours) to train nine technicians. By contrast, skin-snip microscopy training (sample-taking technique and microscopy) took 14 days. Also, supplies, devices and instruments needed for skin-snip microscopy include expensive instruments such as corneoscleral punches, microscopes, microtitre plates, pipettes etc., whereas those for Ov16 RDT principally include the kits from the manufacturer. However, the capital investment of a microscope and Holth corneoscleral punches may not need to be made for each subsequent survey, whereas consumables and Ov16 RDT cassettes would have to be. Although the cost of transport and lodging was considered jointly for the deployment of the two tests and allocated according to the proportion of costs for each specific test and their shared costs, it is important to note that if Ov16 RDT alone were used in typical surveillance, fewer days would likely be necessary, making the Ov16 RDT even less costly. Nevertheless, challenges with Ov16 RDT procurement must be considered when planning surveillance activities.

This study employed the Ov16 RDT using whole blood to obtain results in the field. According to the product insert, the Ov16 RDT demonstrates a sensitivity of 81.1% (95% CI: 70.7%–88.4%) and a specificity of 99.0% (95% CI: 94.8%–99.8%) when compared to skin-snip microscopy as the reference standard [10]. However, collecting dried blood spot (DBS) samples that are eluted in the lab and used in the RDT may reach a sensitivity similar to that of the Ov16 ELISA recommended by the WHO [48,49]. Obtaining high-quality DBS samples, storing and transporting them appropriately to the lab, obtaining eluates and testing these in the laboratory with the Ov16 RDT will certainly increase time and cost [53,54], but this might be an acceptable trade-off between improving the test sensitivity, mitigating the risk of missing infected individuals, and obtaining a reasonable acceptability amongst communities and technicians that should be further investigated. Conducting this work would also follow the recommendation made in [39] that further comparisons of the field performance of the Ov16 RDT be made with the laboratory performance of RDT using DBS, particularly in low-transmission settings (either because of these being hypoendemic—and treatment-naïve—or due to long-term treatment).

### Limitations

Our study has several limitations that should be addressed in future work. Firstly, study participants were not randomly sampled and, therefore, our findings cannot be generalized. Sample sizes to ensure a given power or precision were not calculated before the study; only people who agreed to gather at the focal point in their communities were included in the study (convenience sampling), with only 12% of the population participating overall (Table 1). This likely led to bias. Participants with clinical manifestations seeking to be examined by the research team may also be those with greater levels of infection, leading to overestimation of prevalence levels. Alternatively, individuals who have greater adherence to treatment may also have greater willingness to participate in surveillance activities, with the surveys missing a proportion of those who have higher levels of infection, resulting in underestimation. Our small sample size would also have had an impact on our assessment of diagnostic accuracy. Secondly, social desirability bias may have influenced the results of the usability and acceptability surveys, as they were based purely on verbal responses. Thirdly, the slides prepared from the incubation medium of the skin snips for detection of *O. volvulus* microfilariae were not fixed and stained to assess their characteristic morphological features, and no skin-snip PCR was conducted to confirm species-specific identification. The presence of *M. perstans* has indeed been reported in the middle-belt of Ghana (albeit not in our study area) [55]. Although we cannot totally rule out the presence of microfilariae of other filarial species in the skin snips such as those of *M. perstans* (blood-dwelling) or *M*. *streptocerca* (skin-dwelling), we think it unlikely that this affected our results for the reasons discussed above. Fourthly, we used the Ov16 RDT with whole blood, but collecting DBS samples that are eluted in the lab and used in the RDT may reach a sensitivity similar to that of the Ov16 ELISA recommended by the WHO [53,54]. Finally, as our cost estimates were calculated using specific costs for Ghana, there is a need for similar implementation research in other endemic countries and settings, as exemplified by the very different cost estimates presented for Senegal [12].

## Conclusions

This study has demonstrated the usability of the Ov16 RDT to identify ongoing transmission using seroprevalence in children aged 5–9 years in an area with nearly three decades of CDTI, and more generally, exposure patterns in the general population. The test was also more acceptable and feasible than skin-snip microscopy, less costly to implement, and may help overcome the increasing reluctance of endemic communities to participate in onchocerciasis surveillance activities. Regardless of these advantageous features, further R&D efforts are necessary to increase the diagnostic performance of serological tests and improve their ability to distinguish between past and patent infection [56].

## Supporting information

S1 Text Interviewer guide for participants’ and technicians’ ‘exit’ interviews after conducting tests. (PDF)

S2 Text Breakdown of cost of diagnostic test. (XLSX)

S1 Table Age and sex distribution of anti-Ov16 RDT, skin-snip and palpable nodule positivity. (DOCX)

## Abbreviations

CDTI: Community Directed Treatment with Ivermectin
CI: Confidence interval
DALY: Disability Adjusted Life Year
DBS: Dried blood spot
ELISA: Enzyme-Linked Immunosorbent Assay
EOT: Elimination of Transmission
GBD: Global Burden of Disease
IgG: Immunoglobulin G
IgG4: Immunoglobulin G4
MDA: Mass Drug Administration
NTD: Neglected Tropical Disease
OCP: Onchocerciasis Control Programme in West Africa
PCR: Polymerase Chain Reaction
PES: Post-Elimination Surveillance
PTS: Post-Treatment Surveillance
R&D: Research and Development
REMO: Rapid Epidemiological Mapping of Onchocerciasis
RDT: Rapid Diagnostic Test
SSA: Sub-Saharan Africa
UI: Uncertainty interval
WHO: World Health Organization

## References

[1] World Health Organization. Onchocerciasis. 2022. Available from: https://www.who.int/news-room/fact-sheets/detail/onchocerciasis

[2] Institute for Health Metrics and Evaluation (IHME). Findings from the Global Burden of Disease Study 2017. Seattle, WA: IHME, 2018. Available from: https://www.healthdata.org/sites/default/files/files/policy_report/2019/GBD_2017_Booklet.pdf

[3] GBD 2021 Diseases and Injuries Collaborators. Global incidence, prevalence, years lived with disability (YLDs), disability-adjusted life-years (DALYs), and healthy life expectancy (HALE) for 371 diseases and injuries in 204 countries and territories and 811 subnational locations, 1990-2021: a systematic analysis for the Global Burden of Disease Study 2021. Lancet. 2024;403:2133–61. doi: 10.1016/S0140-6736(24)00757-8 38642570 PMC11122111

[4] Remme JHF, Boatin B, Boussinesq M. Helminthic diseases: onchocerciasis and loiasis. In: Quah SR, Cockerham WC, editors. The international encyclopedia of public health. 2nd ed. Oxford: Elsevier; 2017. pp. 576–8.

[5] Nana-Djeunga HC, Bourguinat C, Pion SD, Bopda J, Kengne-Ouafo JA, Njiokou F, et al. Reproductive status of Onchocerca volvulus after ivermectin treatment in an ivermectin-naïve and a frequently treated population from Cameroon. PLoS Negl Trop Dis. 2014;8(4):e2824. doi: 10.1371/journal.pntd.0002824 24762816 PMC3998936

[6] Bockarie MJ, Kelly-Hope LA, Rebollo M, Molyneux DH. Preventive chemotherapy as a strategy for elimination of neglected tropical parasitic diseases: endgame challenges. Philos Trans R Soc Lond B Biol Sci. 2013;368(1623):20120144. doi: 10.1098/rstb.2012.0144 23798692 PMC3720042

[7] Mutono N, Basáñez M-G, James A, Stolk WA, Makori A, Kimani TN, et al. Elimination of transmission of onchocerciasis (river blindness) with long-term ivermectin mass drug administration with or without vector control in sub-Saharan Africa: a systematic review and meta-analysis. Lancet Glob Health. 2024;12(5):e771–82. doi: 10.1016/S2214-109X(24)00043-3 38484745 PMC11009120

[8] World Health Organization. Ending the neglect to attain the Sustainable Development Goals: a road map for neglected tropical diseases 2021–2030. Available from: https://www.who.int/publications/i/item/9789240010352

[9] Lakwo T, Oguttu D, Ukety T, Post R, Bakajika D. Onchocerciasis elimination: progress and challenges. Res Rep Trop Med. 2020;11:81–95. doi: 10.2147/RRTM.S224364 33117052 PMC7548320

[10] Taylor HR, Munoz B, Keyvan-Larijani E, Greene BM. Reliability of detection of microfilariae in skin snips in the diagnosis of onchocerciasis. Am J Trop Med Hyg. 1989;41:467–71. doi: 10.4269/ajtmh.1989.41.467 2802024

[11] Bottomley C, Isham V, Vivas-Martínez S, Kuesel AC, Attah SK, Opoku NO, et al. Modelling Neglected Tropical Diseases diagnostics: the sensitivity of skin snips for *Onchocerca volvulus* in near elimination and surveillance settings. Parasit Vectors. 2016;9(1):343. doi: 10.1186/s13071-016-1605-3 27301567 PMC4908809

[12] Dieye Y, Storey HL, Barrett KL, Gerth-Guyette E, Di Giorgio L, Golden A, et al. Feasibility of utilizing the SD BIOLINE Onchocerciasis IgG4 rapid test in onchocerciasis surveillance in Senegal. PLoS Negl Trop Dis. 2017;11(10):e0005884. doi: 10.1371/journal.pntd.0005884 28972982 PMC5640270

[13] Abong RA, Amambo GN, Chounna Ndongmo PW, Njouendou AJ, Ritter M, Beng AA, et al. Differential susceptibility of *Onchocerca volvulus* microfilaria to ivermectin in two areas of contrasting history of mass drug administration in Cameroon: relevance of microscopy and molecular techniques for the monitoring of skin microfilarial repopulation within six months of direct observed treatment. BMC Infect Dis. 2020;20(1):726. doi: 10.1186/s12879-020-05444-2 33008333 PMC7530974

[14] Lobos E, Weiss N, Karam M, Taylor HR, Ottesen EA, Nutman TB. An immunogenic Onchocerca volvulus antigen: a specific and early marker of infection. Science. 1991;251(5001):1603–5. doi: 10.1126/science.2011741 2011741

[15] Cama VA, McDonald C, Arcury-Quandt A, Eberhard M, Jenks MH, Smith J, et al. Evaluation of an OV-16 IgG4 enzyme-linked immunosorbent assay in humans and its application to determine the dynamics of antibody responses in a non-human primate model of *Onchocerca volvulus* infection. Am J Trop Med Hyg. 2018;99(4):1041–8. doi: 10.4269/ajtmh.18-0132 30062989 PMC6159584

[16] Solomon AW, Engels D, Bailey RL, Blake IM, Brooker S, Chen J-X, et al. A diagnostics platform for the integrated mapping, monitoring, and surveillance of neglected tropical diseases: rationale and target product profiles. PLoS Negl Trop Dis. 2012;6(7):e1746. doi: 10.1371/journal.pntd.0001746 22860146 PMC3409112

[17] Golden A, Steel C, Yokobe L, Jackson E, Barney R, Kubofcik J, et al. Extended result reading window in lateral flow tests detecting exposure to *Onchocerca volvulus*: a new technology to improve epidemiological surveillance tools. PLoS One. 2013;8(7):e69231. doi: 10.1371/journal.pone.0069231 23935960 PMC3720650

[18] Vlaminck J, Fischer PU, Weil GJ. Diagnostic Tools for Onchocerciasis Elimination Programs. Trends Parasitol. 2015;31(11):571–82. doi: 10.1016/j.pt.2015.06.007 26458784

[19] Biritwum NK, de Souza DK, Asiedu O, Marfo B, Amazigo UV, Gyapong JO. Onchocerciasis control in Ghana (1974-2016). Parasit Vectors. 2021;14:3. doi: 10.1186/s13071-020-04507-2 33388081 PMC7778817

[20] Ghana Health Service. Ghana NTD Master Plan 2016–2020. Expanded Special Project for Elimination of Neglected Tropical Diseases. 2020. Available from: https://espen.afro.who.int/system/files/content/resources/GHANA_NTD_Master_Plan_2016_2020.pdf

[21] Otabil KB, Basáñez M-G, Ankrah B, Opoku SA, Kyei DO, Hagan R, et al. Persistence of onchocerciasis and associated dermatologic and ophthalmic pathologies after 27 years of ivermectin mass drug administration in the middle belt of Ghana. Trop Med Int Health. 2023;28(11):844–54. doi: 10.1111/tmi.13937 37846505

[22] Otabil KB, Ankrah B, Bart-Plange EJ, Donkoh ES, Avarikame FA, Ofori-Appiah FO, et al. Prevalence of epilepsy in the onchocerciasis endemic middle belt of Ghana after 27 years of mass drug administration with ivermectin. Infect Dis Poverty. 2023;12:75. doi: 10.1186/s40249-023-01117-9 37587500 PMC10433588

[23] Otabil KB, Basáñez M-G, Ankrah B, Bart-Plange EJ, Babae TN, Kudzordzi P-C, et al. Non-adherence to ivermectin in onchocerciasis-endemic communities with persistent infection in the Bono Region of Ghana: a mixed-methods study. BMC Infect Dis. 2023;23(1):805. doi: 10.1186/s12879-023-08806-8 37974087 PMC10655298

[24] Kura K, Milton P, Hamley JID, Walker M, Bakajika DK, Kanza EM, et al. Can mass drug administration of moxidectin accelerate onchocerciasis elimination in Africa? Philos Trans R Soc Lond B Biol Sci. 2023;378(1887):20220277. doi: 10.1098/rstb.2022.0277 37598705 PMC10440165

[25] Coalition for Operational Research on Neglected Tropical Disease (COR-NTD). Bench Aid for the Onchocerciasis Ov16 Rapid Diagnostic Test (v.1.2). 2016. Available from: https://www.cor-ntd.org/resources/bench-aid-onchocerciasis-ov16-rapid-diagnostic-test

[26] Nditanchou R, Agyemang D, Dixon R, D’Souza S, Selby R, Opare J, et al. Persistent transmission of onchocerciasis in Kwanware-Ottou focus in Wenchi health district. BMC Infect Dis. 2024;24(1):1156. doi: 10.1186/s12879-024-10071-2 39402497 PMC11475550

[27] Hutubessy R, Chisholm D, Edejer TT-T. Generalized cost-effectiveness analysis for national-level priority-setting in the health sector. Cost Eff Resour Alloc. 2003;1(1):8. doi: 10.1186/1478-7547-1-8 14687420 PMC320499

[28] Bank of Ghana. Exchange rates for 2022. [cited May 28 2025]. Available from: https://www.bog.gov.gh/economic-data/exchange-rate/

[29] World Health Organization & African Programme for Onchocerciasis Control. Guidelines for revising ivermectin treatment boundaries within the context of onchocerciasis elimination. African Programme for Onchocerciasis Control. 2015. Available from: https://apps.who.int/iris/handle/10665/343029

[30] Brown LD, Cai TT, DasGupta A. Interval estimation for a binomial proportion. Stat Sci. 2001;16:101–17.

[31] Bland M. An Introduction to Medical Statistics. Oxford: Oxford University Press; 1991.

[32] Eneanya OA, Koudou BG, Aboulaye M, Elvis AA, Souleymane Y, Kouakou M-M, et al. Progress towards onchocerciasis elimination in Côte d’Ivoire: a geospatial modelling study. PLoS Negl Trop Dis. 2021;15(2):e0009091. doi: 10.1371/journal.pntd.0009091 PMC7875389 33566805

[33] Nyagang SM, Cumber SN, Cho JF, Keka EI, Nkfusai CN, Wepngong E, et al. Prevalence of onchocerciasis, attitudes and practices and the treatment coverage after 15 years of mass drug administration with ivermectin in the Tombel Health District, Cameroon. Pan Afr Med J. 2020;35:107. doi: 10.11604/pamj.2020.35.107.16036 32637005 PMC7321683

[34] Kifle B, Woldemichael K, Nigatu M. Prevalence of Onchocerciasis and Associated Factors among Adults Aged ≥ 15 Years in Semen Bench District, Bench Maji Zone, Southwest Ethiopia: Community Based Cross-Sectional Study. Advances in Public Health. 2019;2019:1–9. doi: 10.1155/2019/7276230

[35] Cama VA, Mendizabal-Cabrera R, de Leon O, White M, McDonald C, Thiele E, et al. Changes in Anti-OV-16 IgG4 Responses to Onchocerciasis after Elimination of Transmission in the Central Endemic Zone of Guatemala. Am J Trop Med Hyg. 2024;110(5):943–50. doi: 10.4269/ajtmh.23-0473 38507804 PMC11066353

[36] Willen L, Basáñez MG, Dvorak V, Veriegh FBD, Aboagye FT, Idun B. Human immune response against salivary antigens of *Simulium damnosum* s.l.: a new epidemiological marker for exposure to blackfly bites in onchocerciasis endemic areas. PLoS Negl Trop Dis. 2021;15:e0009512. doi: 10.1371/journal.pntd.0009512 34157020 PMC8253393

[37] Willen L, Milton P, Hamley JID, Walker M, Osei-Atweneboana MY, Volf P, et al. Demographic patterns of human antibody levels to *Simulium damnosum* s.l. saliva in onchocerciasis-endemic areas: An indicator of exposure to vector bites. PLoS Negl Trop Dis. 2022;16(1):e0010108. doi: 10.1371/journal.pntd.0010108 35020729 PMC8789114

[38] Prost A. Latency period in onchocerciasis. Bull World Health Organ. 1980;58(6):923–5. 6971189 PMC2396000

[39] Ov-16 Meeting Notes. Report of a meeting held at the Neglected Tropical Diseases Support Center, Taskforce for Global Health, 2–3 May 2016. Decatur, GA, USA. Available from: https://www.cor-ntd.org/sites/default/files/content/document/Ov16%20Technical%20Meeting%20Report_May_2016.pdf

[40] Gbakima AA, Nutman TB, Bradley JE, McReynolds LA, Winget MD, Hong Y, et al. Immunoglobulin G subclass responses of children during infection with *Onchocerca volvulus*. Clin Diagn Lab Immunol. 1996;3(1):98–104. doi: 10.1128/cdli.3.1.98-104.1996 8770512 PMC170255

[41] Gass KM. Rethinking the serological threshold for onchocerciasis elimination. PLoS Negl Trop Dis. 2018;12(3):e0006249. doi: 10.1371/journal.pntd.0006249 29543797 PMC5854237

[42] Lobos E, Weiss N, Karam M, Taylor HR, Ottesen EA, Nutman TB. An immunogenic *Onchocerca volvulus* antigen: a specific and early marker of infection. Science. 1991;251(5001):1603–5. doi: 10.1126/science.2011741 2011741

[43] Coulibaly YI, Klion AD. Miscellaneous filariae. In: Ryan ET, Hill DR, Solomon T, Aronson NE, Endy TP, editors. Hunter’s Tropical Medicine and Emerging Infectious Diseases. Elsevier; 2020. pp. 872–7.

[44] Hamley JID, Walker M, Coffeng LE, Milton P, de Vlas SJ, Stolk WA. Structural uncertainty in onchocerciasis transmission models influences the estimation of elimination thresholds and selection of age groups for seromonitoring. J Infect Dis. 2020;221(Suppl 5):S510–8. doi: 10.1093/infdis/jiz674 32173745 PMC7289547

[45] Dolo H, Coulibaly YI, Sow M, Dembélé M, Doumbia SS, Coulibaly SY, et al. Serological Evaluation of Onchocerciasis and Lymphatic Filariasis Elimination in the Bakoye and Falémé Foci, Mali. Clin Infect Dis. 2021;72(9):1585–93. doi: 10.1093/cid/ciaa318 32206773 PMC8096229

[46] Paulin HN, Nshala A, Kalinga A, Mwingira U, Wiegand R, Cama V, et al. Evaluation of Onchocerciasis Transmission in Tanzania: Preliminary Rapid Field Results in the Tukuyu Focus, 2015. Am J Trop Med Hyg. 2017;97(3):673–6. doi: 10.4269/ajtmh.16-0988 28722619 PMC5590590

[47] Mshana MI, Silvestri V, Mushi V, Bonaventura WM, Tarimo D, Ngasala B, et al. Burden and factors associated with onchocerciasis transmission among school-aged children after more than 20 years of Community Directed Treatment with Ivermectin in Ulanga district, Tanzania: A school-based cross-sectional study. PLOS Glob Public Health. 2023;3(5):e0001919. doi: 10.1371/journal.pgph.0001919 37172010 PMC10180657

[48] Siewe Fodjo JN, Tatah G, Tabah EN, Ngarka L, Nfor LN, Chokote SE, et al. Epidemiology of onchocerciasis-associated epilepsy in the Mbam and Sanaga river valleys of Cameroon: impact of more than 13 years of ivermectin. Infect Dis Poverty. 2018;7:114. 30501640 10.1186/s40249-018-0497-1 PMC6276171

[49] Barbazan P, Escaffre H, Mbentengam R, Boussinesq M. Entomologic study on the transmission of onchocerciasis in a forest-savanna transition area of Cameroon. Bull Soc Pathol Exot. 1998;91(2):178–82. 9642481

[50] Siewe Fodjo JN, Vieri MK, Ngarka L, Njamnshi WY, Nfor LN, Mengnjo MK, et al. “Slash and clear” vector control for onchocerciasis elimination and epilepsy prevention: a protocol of a cluster randomised trial in Cameroonian villages. BMJ Open. 2021;11(9):e050341. doi: 10.1136/bmjopen-2021-050341 34475178 PMC8413955

[51] Otabil KB, Gyasi SF, Awuah E, Obeng-Ofori D, Tenkorang SB, Kessie JA, et al. Biting rates and relative abundance of *Simulium* flies under different climatic conditions in an onchocerciasis endemic community in Ghana. Parasit Vectors. 2020;13(1):229. doi: 10.1186/s13071-020-04102-5 32375902 PMC7204027

[52] Raimon S, Lakwo TL, Sebit WJ, Siewe Fodjo JN, Alinda P, Carter JY, et al. “Slash and Clear”, a Community-Based Vector Control Method to Reduce Onchocerciasis Transmission by *Simulium sirbanum* in Maridi, South Sudan: A Prospective Study. Pathogens. 2021;10(10):1329. doi: 10.3390/pathogens10101329 34684277 PMC8538802

[53] Nana-Djeunga HC, Sicard CM, Mogoung-Wafo AE, Chesnais CB, Deléglise H, Touka-Nounkeu R. Changes in onchocerciasis Ov16 IgG4 rapid diagnostic test results over one-month follow-up: lessons for reading timeframe and decision-making. Am J Trop Med Hyg. 2022;107:658–61. doi: 10.4269/ajtmh.21-1201 35914689 PMC9490668

[54] Peck RB, Golden AL. Assay of dried blood spots using the SD BIOLINE Onchocerciasis IgG4 Rapid Test. Seattle, WA USA: PATH; 2019. Available from: https://www.path.org/our-impact/resources/assay-dried-blood-spots-using-sd-bioline-onchocerciasis-igg4-rapid-test/

[55] Debrah LB, Nausch N, Opoku VS, Owusu W, Mubarik Y, Berko DA, et al. Epidemiology of *Mansonella perstans* in the middle belt of Ghana. Parasit Vectors. 2017;10(1):15. doi: 10.1186/s13071-016-1960-0 28061905 PMC5219801

[56] Bennuru S, Oduro-Boateng G, Osigwe C, Del Valle P, Golden A, Ogawa GM, et al. Integrating Multiple Biomarkers to Increase Sensitivity for the Detection of *Onchocerca volvulus* Infection. J Infect Dis. 2020;221(11):1805–15. doi: 10.1093/infdis/jiz307 31201416 PMC7213562

