# Supplementary material for: Usability, acceptability, and cost of the SD BIOLINE Ov16 rapid diagnostic test for onchocerciasis surveillance in endemic communities in the middle belt of Ghana
Source: PLoS Negl Trop Dis. 2025 Aug 29;19(8):e0012191. doi: 10.1371/journal.pntd.0012191 (PMC12413087; doi:10.1371/journal.pntd.0012191)
Supplement: S1 Table — (DOCX) [file pntd.0012191.s003.docx]

**S1 Table. Age and sex distribution of test positivity for Ov16 RDT, skin-snip microscopy and nodule palpation for *Onchocerca volvulus* in the study communities, Bono Region, Ghana**

| Category | Positivity by Ov16 RDT  n +ve/N examined  (%) [95% CI] | Positivity by skin-snip  n +ve/N examined  (%) [95% CI] | Positivity by nodule palpation  n +ve/N examined  (%) [95% CI] |
| --- | --- | --- | --- |
| Age^†^ (years) |  |  |  |
| 5–9 | 3/27 (11.1%) [3.9% – 28.1%] | 3/13 (23.1%) [8.2% – 50.3%] | 1/27 (3.7%) [0.7% – 18.3%] |
| 10–19 | 2/36 (5.6%) [1.5% – 18.1%] | 2/15 (13.3%) [3.7% – 37.9%] | 2/36 (5.6 %) [1.5% – 18.1%] |
| 20–29 | 8/51 (15.7%) [8.2% – 28.0%] | 2/16 (12.5%) [3.5% – 36.0%] | 1/51 (2.0%) [0.4% – 10.3%] |
| 30–39 | 11/32 (34.4%) [20.4% – 51.7%] | 3/10 (30.0%) [10.8% – 60.3%] | 2/32 (6.3%) [1.7% – 20.2%] |
| ≥40 | 31/96 (32.3%) [23.8% – 42.2%] | 1/37 (2.7%) [0.5% – 13.8%] | 8/96 (8.3%) [4.3% – 15.6%] |
| Total | 55/242 (22.7%) [17.9% – 28.4%] | 11/91 (12.1%) [6.9% – 20.4%] | 14/242 (5.8%) [3.5% – 9.5%] |
| Sex^ǂ^ |  |  |  |
| Female | 43/136 (31.6%) [24.4% – 39.9%] | 6/53 (11.3%) [5.3% – 22.6%] | 6/136 (4.4%) [2.0% – 9.3%] |
| Male | 17/117 (14.5%) [9.3% – 22.0%] | 5/41 (12.2%) [5.3% – 25.5%] | 8/117 (6.8%) [3.5% – 12.9%] |
| Total | 60/253 (23.7%) [18.9% – 29.3%] | 11/94 (11.7%) [6.7% – 19.8%] | 14/253 (5.5%) [3.3% – 9.1%] |

^†^ Age was recorded for 242 of the 254 participants. ^ǂ^ Sex was recorded for 253 participants.
